# Novel approach to decision making for orphan drugs

**DOI:** 10.1017/S0266462323000053

**Published:** 2023-02-07

**Authors:** Barbora Decker, Tomas Mlcoch, Anastasie Pustovalova, Tomas Dolezal

**Affiliations:** 1 Department of Pharmacology, Faculty of Medicine, Masaryk University, Brno, Czechia; 2 Value Outcomes, Prague, Czechia; 3 Cogvio, Prague, Czechia

**Keywords:** orphan drugs, rare diseases, reimbursement, health policy, health technology assessment

## Abstract

**Background:**

Out of 185 orphan medicinal products (OMPs) registered in 2015–2021, a mere 110 (59 percent) were available to Czech patients, and only 54 (29 percent) were officially reimbursed. Moreover, this proportion has steadily decreased over time. After years of public debate induced by this unsatisfactory OMP patient access, the national viewpoint shifted toward creating a special pathway for the reimbursement of OMP. Thus, a rigorous pricing and reimbursement procedure with strict timelines and elaborated methodology has been recently adopted in Czechia.

**Methodology:**

The innovative legislation follows the recommendations for value assessment and funding processes for rare diseases and incorporates additional elements of value, such as the societal perspective. First, the application with clinical evidence, cost-effectiveness, and budget impact analyses is submitted to the governmental health technology assessment (HTA) agency by the Marketing Authorization Holder or a Health Insurance Fund. Moreover, professional associations and patients’ organizations are rightful participants in the proceeding, providing evidence and comments. Then, the HTA agency performs the assessment/appraisal of the evidence. It subsequently publishes the assessment report summarizing available information. The report is then forwarded to the Ministry of Health and its advisory body consisting of patients, clinical experts, health insurance funds, and the State. They critically evaluate the documents and issue a binding opinion following prespecified decision-making criteria. Based on this binding opinion, the decision is issued by the HTA agency. Thus, the role of the advisory body in this process is crucial.

**Conclusion:**

We believe that this novel approach may offer satisfactory patient access to orphan drugs. Moreover, it serves as a real-world example of “value-based” decision making.

## Background

The European Union (EU) regulation on orphan medicinal products (OMPs) (Regulation No. 141/2000) (1) granted a unified EU approach to orphan drug designation and marketing authorization; it ensured 10-year marketing exclusivity (with a possible 2-yr extension for pediatric indications) or fee waivers (2). Even though the OMP registration process has been harmonized across all EU member states, the pricing and reimbursement (P&R) processes remain fragmented and unpredictable (3), impeding patient access to treatment (2;4).

The assessment of OMP must reflect the characteristics of rare diseases. Typical difficulties stem from the limited experience with the disease and small population of affected individuals, which results in significant uncertainty regarding clinical outcomes (5). The limited number of patients eligible for treatment also severely limits the market potential and consequently raises the price of OMP to cover research costs. Thus, the cost-effectiveness willingness-to-pay (WTP) thresholds are seldom fulfilled to ensure adequate return on investment for pharmaceutical companies (3;6).

In the past few years, considerable attention has been paid to designing general recommendations for value assessment and funding processes for rare diseases (ORPH-VAL) (4). In addition, an ISPOR Special Task Force recently redefined elements of value that should be incorporated in value assessments, in addition to those conventionally included and considered (7). Inspired by this recent scientific research, Czechia designed a unique and innovative P&R strategy to reflect current best practices. The resulting health policy approach is a real-world application of the proposed recommendations and can inspire other countries.

Several publications have described OMP access in specific countries in the Central–Eastern European (CEE) region (8;9) or surveyed a larger number of countries in Europe (3). However, Czechia has scarcely been investigated, and due to the adoption of new orphan legislation in 2022, the prior information is no longer valid.

This paper presents a unique and innovative P&R process for OMP, as stated in Act No. 48/1997 Coll. (10). The description is preceded by a thorough analysis of OMP availability and an explanation of the rationale behind the policy design. The novel approach takes into account specific aspects of rare diseases, assesses OMP value from the societal perspective, and includes patient representatives in the assessment and appraisal process.

## Previous OMP reimbursement process and unmet need

**Figure 1.**
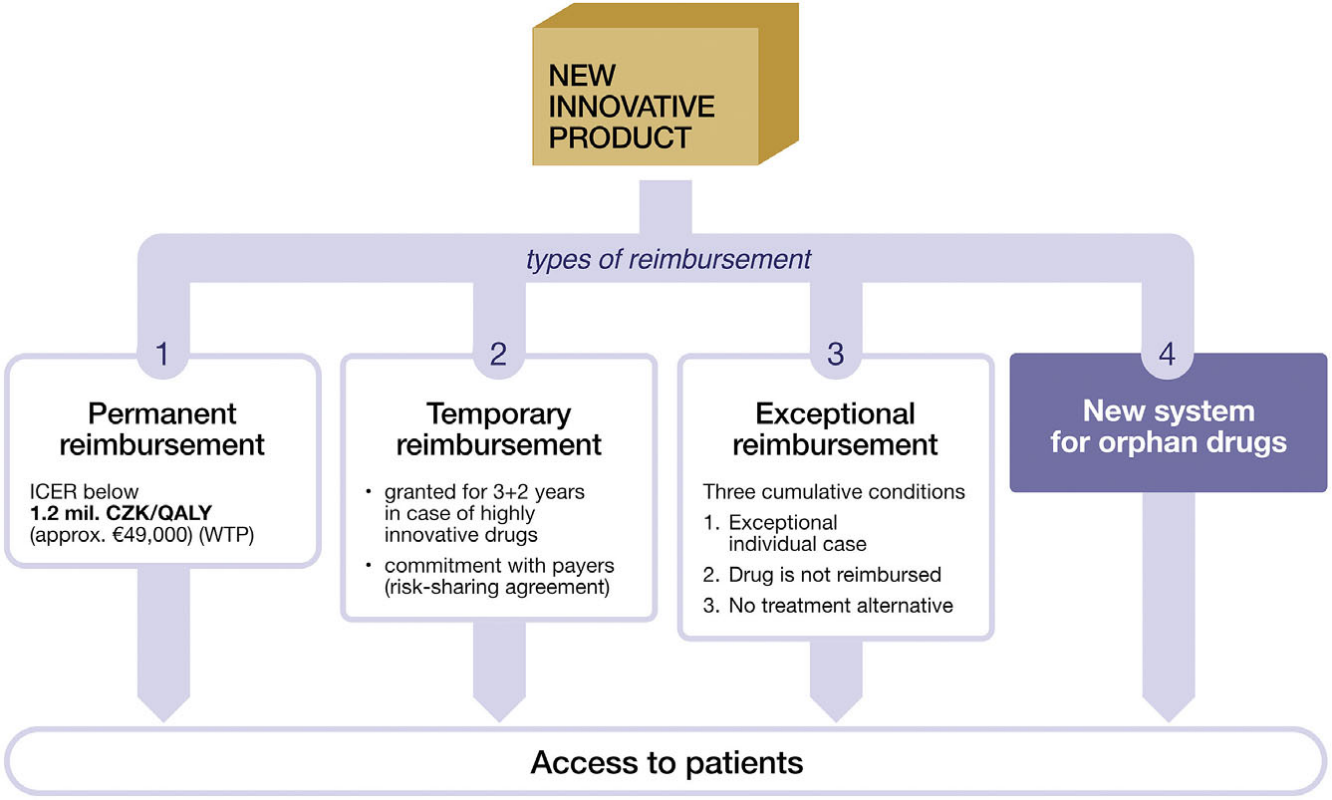
Reimbursement in Czechia.

Many countries still do not have any specific P&R process for OMP (3). Until recently, this was also the case of Czechia, where patient access was usually granted by non-orphan-specific drug programs, that is, temporary or exceptional reimbursement (Figure 1). The situation was too complex, time-consuming, and uncertain for drug suppliers (11;12). Since the external price referencing system pushes prices to the lowest acceptable level, pharmaceutical companies often completely avoid or at least postpone entering the Czech market due to the consecutive price-drop effect in other countries.

**Figure 2.**
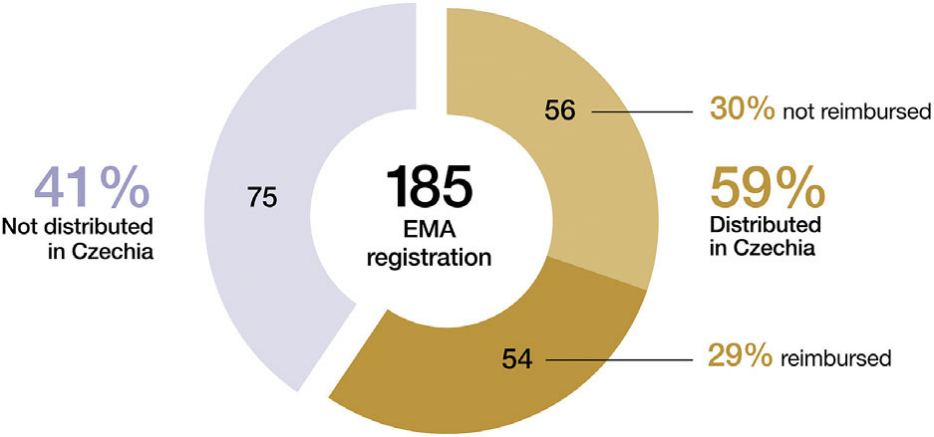
Availability of OMP (2015–2021).

Therefore, out of 185 OMPs registered by European Medicines Agency (EMA) from 2015 to 2021, a mere 110 (59 percent) were available to Czech patients, and only 54 (29 percent) were officially reimbursed (Figure 2) (13–15). Moreover, the proportion of officially reimbursed OMP has steadily decreased over time (16).

The situation is even worse in other countries in the CEE region, with the proportion of reimbursed OMP varying from 6.3 percent in Latvia to 27.4 percent in Poland, as shown in the analysis by Malinowski et al. (17). On the other hand, the proportion of OMP reimbursed in Western-European countries varies from 33 to 93 percent (18;19). Orphan drug availability is thus more restricted in lower-income European countries with budget limitations (20).

It is clear that even though therapies are available for many rare conditions, access to care is often heavily restricted. Thus, Czechia had to respond to this urgent public health issue by updating the decision-making process and the whole legal framework to provide safe and effective therapies for patients suffering from rare diseases.

## The new national pricing and reimbursement strategy

After years of debate induced by unsatisfactory OMP access, the national viewpoint shifted toward creating a special status for the reimbursement of OMP. The updated legislation followed the ORPH-VAL recommendations (4), for example, assessing the OMP value on patient/healthcare system/societal level, including its uncertainty. The methodology of including these principles in the P&R procedure is described in detail.

**Figure 3.**
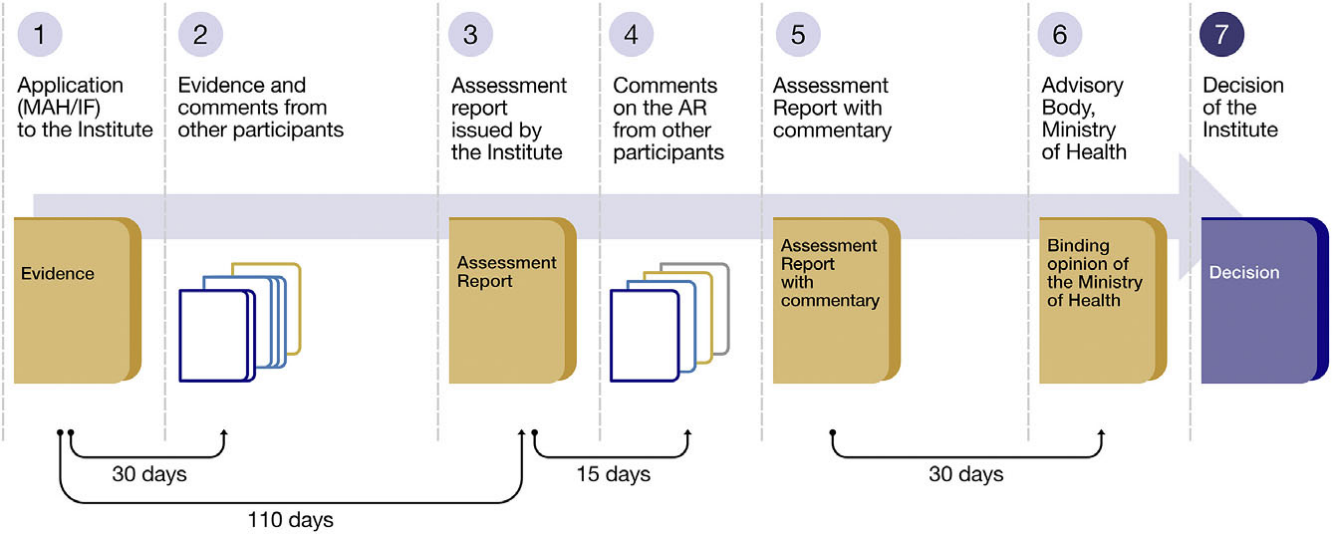
Schematic of the administrative procedure.

The initial appraisal/assessment of OMP is performed by the governmental health technology assessment (HTA) agency, that is, the State Institute for Drug Control (“the Institute”). It critically reviews and assesses the clinical and economic evidence, then formulates its opinion in the form of an assessment report. The complete process is depicted in Figure 3.

[1] The application with clinical and cost-effectiveness evidence and budget impact analyses is submitted to the Institute by the Marketing Authorization Holder (MAH) or by one of the health insurance funds; thus, initiating the proceeding.

[2] In addition to the MAH and insurance funds, the relevant professional associations and relevant patients’ organizations also participate in the administrative proceeding. Therefore, they are entitled to present evidence and make comments during the 30 days after the initiation of administrative proceedings. This ensures the essential involvement of healthcare professionals and affected patients in the P&R process.

[3] Within 110 days from the initiation of the proceedings, the Institute shall issue an assessment report summarizing available information on the efficacy and safety of the OMP, the disease for which it is indicated, the current treatment of the disease, and the effects of treatment on the patient’s quality of life (QoL), the impact on health insurance and social security systems, and/or the overall impact from a societal perspective.

For this purpose, the Institute is entitled to request any necessary information from the Institute of Health Information and Statistics, the Czech Statistical Office, public health insurance funds, relevant social security bodies, relevant medical professional societies, and patient organizations.

[4] All the participants have the right to comment on the assessment report within 15 days from its publication.

[5] The Institute then publishes the final assessment report and forwards it together with a summary of the participants’ statements and possible managed entry agreements (MEAs) to the Ministry of Health and its advisory body (described further in the article).

[6] Based on these documents, the ministry should issue a binding opinion within 30 days. The binding opinion results from discussions within the advisory body established beforehand by the Minister of Health.

The advisory body consists of four stakeholders: (i) patients (not with the given disease, i.e., from a different patient organization), (ii) clinical experts (not from the given disease area), (iii) public health insurance funds, and (iv) the State. Each stakeholder has two representatives, and the binding opinion has to be agreed to by the majority of voters (five out of eight votes). The advisory body members are appointed and dismissed by the Minister of Health with the term of office set to 3 years. A member of the advisory body can be reappointed repeatedly. Proposals for the appointment and dismissal of members of the advisory body are submitted to the Minister of Health by (i) the Patient Council of the Minister of Health after consultation with the Czech Association for Rare Diseases, (ii) the Czech Medical Society of Jan Evangelista Purkyně, (iii) public health insurance funds, and (iv) the Deputy Minister for Economics and Health Insurance, respectively. At least four representatives are nominated for each sector. The nomination shall be accompanied by a written declaration of the nominee’s conflict of interest. In the case of medical society or patients, the nominees should be members of a different medical society or patient organization (i.e., not related to the assessed condition due to conflict of interest). From these nominees, the Minister of Health chooses two representatives for each stakeholder, that is, eight in total. The members shall have no conflict of personal or professional interests, and they shall not misuse any information obtained through their service on the advisory body.

Evaluation criteria specified by law and assessed by the advisory body are summarized in Table 1. These criteria are in line with other multicriteria decision analyses (MCDA) criteria used to evaluate orphan drugs (21) and with the ORPH-VAL framework (4). There are no specific value thresholds or weights for individual criteria, but a summary of each criterion’s justification and anonymous voting results shall be publicly available. This ensures the transparency and legitimacy of the decision and the whole process.

**Table 1.** Criteria and parameters for OMP assessment (according to Order n. 53/2021 from the Minister of Health)

Evaluated criteria	Methodology	Criteria for decision
(a) Therapeutic effectiveness (1) and safety (2)	(i) Effect on survival, morbidity, QoL, or other significant clinical outcomes (ii) Severe adverse events profile, the occurrence of adverse events leading to treatment discontinuation	(i) Prioritize OMP with significant efficacy on major clinical outcomes (survival, QoL, complications, hospitalizations, long-term disability), with regard to the level of clinical evidence (incl. RWE) and corresponding level of uncertainty (ii) Prioritize OMP with significant improvement in safety profile in case Standard of Care (SoC) toxicity is a major limitation
(b) Severity of disease	Expected life expectancy without treatment, QoL, incidence of (irreversible) complications	Prioritize OMP for diseases that severely decrease life expectancy and/or QoL without treatment
(c) Reimbursed treatment alternatives	Description of the current treatment algorithm	Prioritize OMP indicated for rare diseases with no treatment alternative
(d) Societal impact	(i) Costs assessed from the societal perspective, including loss of productivity (ii) Dependency of others – family, caregivers, need for home-care, long-term hospitalization, or institutionalization	(i) Prioritize OMP reducing costs from the societal perspective, including indirect costs (loss of productivity, social care costs) (ii) Prioritize OMP, decreasing family/caregiver/societal burden
(e) QoL	Treatment effect on the patient’s QoL	Prioritize OMP with robust evidence, ideally measured in clinical studies
(f) Network of specialized medical centers	Existing network of healthcare providers and diagnostic tools	Provision of effective continuous care delivered by qualified healthcare professionals
(g) Clinical guidelines	Nationally and internationally recognized clinical guidelines relevant for the OMP	Prioritize treatment included in the guidelines, with a high level of evidence and/or grade of recommendation
(h) MEAs with payers	Proposed managed entry scheme (simple discount, budget cap and pay-back, price–volume or outcomes-based agreement)	Prioritize outcome-based models where the manufacturer covers costs associated with ineffective treatment (outcome guarantees)
(i) Cost-effectiveness	Costs per QALY critically assessed by the Institute, absolute QALYs gain	
(j) Budget impact	Healthcare payers costs using a 5-yr time horizon	Prioritize OMP delivering high benefit with acceptable budget impact

Abbreviations: MEAs, managed entry agreements; OMP, orphan medicinal products; QALYs, quality-adjusted life-years; QoL, quality of life; RWE, real-world evidence.

[7] The binding opinion is then forwarded back to the Institute, which then issues a final decision on the P&R in line with the opinion. If the applicant disagrees with the conditions proposed by the binding opinion, the Institute will not grant reimbursement from health insurance funds. In case of a negative binding opinion, the applicant may submit a new application no earlier than 6 months after the administrative proceeding.

After 3 years, the Institute shall re-examine the maximum price according to the current external reference prices. Moreover, after at least 1 year, a health insurance company or the Institute can apply for a reassessment of the reimbursement price for the OMP in case it exceeds the estimated budget impact, there is a change in effectiveness, safety in clinical practice did not meet the preconditions set for reimbursement, or the relevant guidelines have changed. If the Institute cancels the reimbursement, the health insurance company is obliged to reimburse additional treatment costs for up to 12 months. The option of future reassessment decreases the uncertainty and offers the possibility of changing the decision in light of new evidence.

## Other P&R strategies

Figure 1 presents four types of reimbursement in Czechia, formulated in Act No. 48/1997 Coll. and related decrees (10).

[1] For permanent reimbursement, the use of medicinal products needs to be cost-effective with a WTP threshold of 1.2 million CZK per QALY (approx. €49,000). The threshold was defined in relation to the incremental cost-effectiveness ratios for already reimbursed technologies and corresponded to approximately three times the gross domestic product per capita (22). This particular WTP threshold is relatively generous compared to other CEE as well as several western countries (e.g., UK) but is still generally too high for OMP. Moreover, OMP are generally unable to prevail in the rigorous evaluation process (23) due to the inherent clinical/economic uncertainty.

[2] The temporary reimbursement for highly innovative drugs (HIDs) was previously described by Ornstova et al. (24) and Vostalova et al. (12). However, the HID program has changed slightly, together with the new OMP legislation. This type of reimbursement is now granted for a minimum of 3 years and can be prolonged for another 2 years without the need of being cost-effective below a fixed WTP threshold. Then, the product must transition to permanent reimbursement with the fulfillment of its rigorous cost-effectiveness criteria. Nevertheless, in the case of OMPs, the uncertainty and high costs usually last for the whole 5-year period, and the strict cost-effectiveness criteria still cannot be met. This scheme is therefore not attractive or widely acceptable for OMP.

Furthermore, the innovative product has to fulfill at least one of the HID criteria, that is, (a) the primary endpoint in the clinical study demonstrated at least a 30 percent improvement compared to SoC and the endpoint must affect QoL, or (b) the innovative product increases the median overall survival by at least 30percent and by a minimum of 3 months compared to standard of care. For OMP, the evidence is usually not strong enough to meet these criteria since there is often limited comparative data and overall uncertainty.

Another drawback compared to permanent reimbursement **[1]** is the enforced budget cap for the entire temporary reimbursement period and the mandatory risk-sharing agreement. Nevertheless, the orphan pathway also strongly favorites applications with a MEA proposal. The applicable MEAs are simple discount, budget cap, pay-back, price–volume, or outcomes-based agreement (Table 1).

Another disadvantage of temporary reimbursement is the risk linked to re-evaluating the “highly innovative status” if another drug enters permanent reimbursement with the same indication. However, this usually is not the case with OMP since multiple products are generally not in development at the same time.

[3] If the medicinal product is not permanently reimbursed, in cases where it is the only treatment available, the product can still be covered based on an individual patient request (Section 16 of the Act No. 48/1997 Coll., on Public Health Insurance (10)). This exceptional reimbursement is assessed individually on a case-by-case basis, and it is only valid for 3 months, after which the application has to be resubmitted. This puts a substantial bureaucratic burden both on patients and healthcare professionals as well those assessing the application. Generally, continuous patient access to the product cannot be granted through this application process since it is unpredictable, burdensome, and generally obscure.

It is necessary to stress that the HTA process in Czechia is currently implemented only for pharmaceuticals used in outpatient (ambulatory) care. The reimbursement of inpatient drugs used in hospital care lacks full transparency. Hospital budgets cover these drugs without a transparent decision-making process and public oversight. Only two stakeholders are involved in the process, that is, hospitals and health insurance funds, with no representation from other healthcare professionals, professional associations, or patients; additionally, it lacks a thorough and transparent HTA process.

## Conclusions

The most convenient and specific way to access OMP is for them to gain a standard permanent reimbursement with the price fulfilling the WTP threshold (EUR 49,000 per QALY). Thus, the major strength of the Orphan drug legislation is the loosening of the WTP threshold, allowing OMP (with naturally higher Incremental Cost-Effectiveness Ratio (ICER)) to enter the healthcare system with an agreed patient access scheme, clear indication criteria, and funding. Thus, they do not bypass the healthcare system. We can also expect less restrictive budget caps and discount requirements from healthcare payers compared to standard reimbursement pathway.

Another crucial innovation in OMP appraisal is the change in perspective. The OMP value is assessed from the perspective of patients, the healthcare system as well as the wider society. This is ensured by the involvement of patient organizations as well as healthcare professionals in the procedure and by incorporating the societal perspective into the evaluated criteria (i.e., impact on patients, burden of disease, cost-effectiveness, and budget impact analyses). A specific and practical feature of this policy is the involvement of patient representatives in the process as lawful members of the advisory body. In this manner, the new strategy fosters multi-stakeholder dialogue and consensus.

Finally, the orphan legislation reflects the newest scientific research derived from rigorous and proven methodologies (4;7).

One of the limitations is the necessity of a valid orphan designation from the EMA during the whole administrative proceeding. If orphan designation status expires, the manufacturer can no longer apply. This can create a barrier for older orphan drugs that continue to provide significant benefits to patients but have high prices, as they can no longer use this reimbursement pathway.

Moreover, the MAH is usually forced to propose a MEA. However, the role of MEA is crucial in OMP assessment since they help to manage the uncertainty associated with the introduction of OMP (25). Moreover, it is favorable from the perspective of budget planning and sustainability of the whole healthcare system.

It is also important to note that in cases where reimbursement is provided at the request of the MAH and the OMP costs exceed the amount presented in the budget impact analyses, the MAH will reimburse the overbudget costs. This, again, might be considered a strength from the perspective of budget planning and financial predictability of the future costs.

Finally, a permanent reimbursement is not granted “forever” since it is possible to re-evaluate it after at least a year and reassess any uncertainties in the decision. However, this can be viewed positively from the perspective of the entire system since it allows faster access and lowers the long-term uncertainty of the decision.

Considering all the benefits and drawbacks, we firmly believe that the described policy is fit for the purpose. There is no doubt that without special conditions for OMP, pharmaceutical companies lack the incentive to invest in OMP research.

The key policy recommendation that could be used in other countries is the incorporation of a value-based framework into decision making, assessing not only clinical effectiveness/safety, cost-effectiveness, and budget impact, but also proposed MEAs, societal impact, and other aspects that adequately reflect the OMP value. Incorporating a value-based framework can help to ensure that decisions about OMP reimbursement are made in a holistic and transparent manner, and that all relevant factors are considered. It can also help to ensure that the value of OMP is adequately reflected in the reimbursement process and that the interests of all stakeholders are taken into account.

## Data Availability

The datasets used and analyzed during the current study are available from the corresponding author on request.
